# A pan-CRISPR analysis of mammalian cell specificity identifies ultra-compact sgRNA subsets for genome-scale experiments

**DOI:** 10.1038/s41467-022-28045-w

**Published:** 2022-02-02

**Authors:** Boyang Zhao, Yiyun Rao, Scott Leighow, Edward P. O’Brien, Luke Gilbert, Justin R. Pritchard

**Affiliations:** 1 Department of Biomedical Engineering, Pennsylvania State University, State College, PA USA; 2 Quantalarity Research Group, Houston, TX USA; 3 Huck Institute for the Life Sciences, Pennsylvania State University, State College, PA USA; 4 Department of Chemistry, Pennsylvania State University, State College, PA USA; 5 Department of Urology, University of California at San Francisco, San Francisco, CA USA; 6 Department of Cellular & Molecular Pharmacology, University of California at San Francisco, San Francisco, CA USA; 7 Helen Diller Family Comprehensive Cancer Center, San Francisco, San Francisco, CA USA

**Keywords:** Genetic engineering, Genomic engineering, Machine learning, Functional genomics

## Abstract

A genetic knockout can be lethal to one human cell type while increasing growth rate in another. This context specificity confounds genetic analysis and prevents reproducible genome engineering. Genome-wide CRISPR compendia across most common human cell lines offer the largest opportunity to understand the biology of cell specificity. The prevailing viewpoint, synthetic lethality, occurs when a genetic alteration creates a unique CRISPR dependency. Here, we use machine learning for an unbiased investigation of cell type specificity. Quantifying model accuracy, we find that most cell type specific phenotypes are predicted by the function of related genes of wild-type sequence, not synthetic lethal relationships. These models then identify unexpected sets of 100-300 genes where reduced CRISPR measurements can produce genome-scale loss-of-function predictions across >18,000 genes. Thus, it is possible to reduce in vitro CRISPR libraries by orders of magnitude—with some information loss—when we remove redundant genes and not redundant sgRNAs.

## Abstract (web summary)

Context specificity confounds genetic analysis and prevents reproducible genome engineering. Here, the authors report a pan-CRISPR analysis of specificity in mammalian cells and identify ultra-compact sgRNA subsets for genome-scale screens.

## Introduction

Large-scale transcriptomics atlases have identified thousands of ubiquitously expressed mammalian genes^1^. Single-cell transcriptomics can rapidly enumerate the differential expression of the genome across a tissue^2,3^. Yet this unprecedented resolution in transcriptomics fails to tell us how genes function in different cell types. Importantly, while transcriptomics easily scales to measure thousands of transcripts in a single cell, functional genomics can only measure the phenotypic effect of a single genotype in a single cell. The knockout of a single gene can cause lethality in one cell and no discernible phenotype in another^4^. This diversity is even known to exist across KRAS mutant cell lines. Some G12X mutated cells are sensitive to loss of KRAS while others aren’t^5^. Moreover, in biotechnology applications, strategies to engineer mammalian cell lines to withstand the stress of stirred-tank bioreactors are not reproducible across cell types^6–9^. These phenotypic differences constitute a poorly understood, but a well-recognized phenomenon called context specificity that is important in cell biology, genetics, biotechnology, and medicine^10^. Context-specific observations are a classic problem in mammalian cell biology and genetics that dates to the origins of the field^11^. Understanding them aids our understanding of basic biological and genomic processes. It also improves our potential to guide those processes rationally.

However, understanding cell specificity means contending with the fact that mammalian cell lines span an incredible diversity of potentially relevant contexts that include; nucleotide mutations, chromosomal abnormalities, copy number alterations, transcriptional profiles, and tissues of origin^12^. Together, these contexts drive the richness and the confusion of mammalian cell biology/genetics. CRISPR screening efforts by the Sanger and Broad institutes have performed genome-wide loss-of-function studies in ~1000 unique mammalian cell lines^4,13^. These studies have identified extensive phenotypic diversity (as measured by differences in CERES scores) in many genes^13,14^. Synthetic lethality has been the dominant paradigm that has been used to explore cell-type-specific sensitivity to loss-of-function in mammalian cell lines. Classic synthetic lethality occurs when a specific somatic mutation confers sensitivity to genetic or chemical-genetic loss-of-function^15^. This is best exemplified by the loss-of-function mutations in BRCA1/2 that predict exquisite sensitivity to the loss of PARP1/2. This relationship has led to the clinical application of PARP inhibitors in cancer^16^. Beyond this paradigmatic example, a number of interesting versions of synthetic lethality have been suggested, including; dosage lethality, paralog lethality, and lineage-specific sensitivities^16–21^. All of these have been useful ideas to investigate the origins of cell-type specificity and to create potential biomarkers for cancer therapy. However, the number of new and exciting synthetic lethality relationships discovered appears to be smaller than the phenotypic diversity observed in large-scale cell-line studies^10^, indicating that there is room for improvement in current association datasets, especially when we broaden our view to consider all applications of mammalian cell lines in basic science and biotechnology.

One likely additional explanation for context specificity is that some molecular markers remain unmeasured. For instance, a yet unmeasured predictive marker is a potential explanation for any selective essential phenotype without a clear explanation. A second explanation is a lack of statistical power to identify rare variants or variants with modest effect sizes across large and heterogeneous groups of cells. Both explanations can be tested and addressed by examining more data across more cell lines. But, beyond these typical explanations, we posit that the static genomic features of unperturbed cell lines may not be sufficient to predict context specificity. CRISPR knockouts create dynamic measurements of cellular responses to gene loss. This dynamic information may be useful to understand context specificity.

In this study, we explore the origins of cell-type specificity as a question of basic mammalian cell biology and genetics to enable rational genome editing. We seek basic genetics understanding and an enhanced ability to design cells for biotechnology applications. To do this, we take a data-driven approach that focuses on predicting cell-type specificity with machine-learning models. We carefully build thousands of machine-learning models that incorporate the effects of millions of CRISPR knockout phenotypes in addition to mutations, copy number, lineage, and RNA-seq to predict cell-type-specific phenotypes. Our analysis reveals that the best models of cell-type-specific CRISPR loss-of-function phenotypes are composed of other CRISPR loss-of-function phenotypes in a pooled library. Furthermore, predictive CRISPR features fall into highly clustered and cross-predictive subnetworks. Inspired by the ideas behind data compression and previous work in transcriptomics^22^, we propose an approach for dramatically compressing genome-scale CRISPR functional genomics experiments. In lossy compression, orders of magnitude reductions in file sizes are exchanged for some acceptable tradeoff in data quality. Instead of in silico data, our models can compress in vitro CRISPR library composition. They identify reduced sets of CRISPR constructs that can predict the loss-of-function effects of unmeasured genes at tunable scales.

## Results

### Cell-type-specific loss-of-function phenotypes are predictable with machine learning

Recent efforts at the Broad and Sanger institutes have created an unprecedented resource to investigate the origins of context-specific dependencies across mammalian cell lines. Context-specific behavior in CRISPR loss-of-function phenotypes can be defined across cell lines using CERES scores^13^. Briefly, a CERES score measures the size of the fitness difference that is observed in a pooled screen from a single gene in a single mammalian cell line. CERES scores are calculated for every gene in the library and they account for multiple confounding effects that bias the direct measurements of individual sgRNA enrichment and depletion. Negative CERES scores denote slower growth, and positive scores denote faster growth. Thus, the set of CERES scores for a given gene across all cell lines provides a high-resolution measurement of cell-type specificity.

**Fig. 1:**
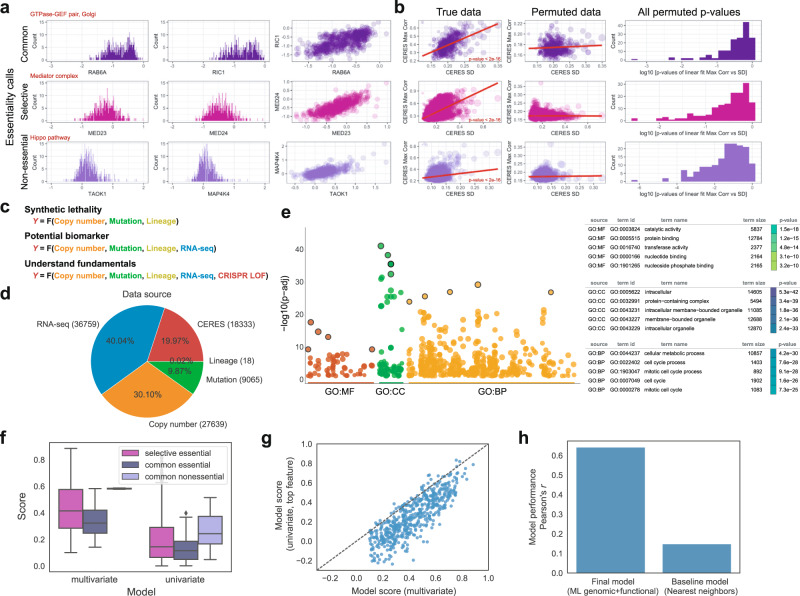
Multivariate machine-learning models predict context specificity. **a** Examining the largest and most cell-type-specific genes in the DepMap dataset identified genes for whom CERES scores were highly correlated with a biologically related gene in the same pathway or complex. This was true in common, selective, and nonessential DepMap genes. This suggested that continuous variation in CERES scores can have biological meaning. **b** Scatter plots (left, middle) have 18,333 unique genes as data points. On *x* axis, the standard deviation of CERES score for each gene; on *y* axis, the maximum correlation from all pairwise Pearson correlations for each gene to all other genes. The point size is proportional to the range of CERES scores across all cell lines. The most cell-type-specific genes tend to have the strongest correlations with CERES scores in other genes. This is measured via a linear model (red line), and the *P* value of this model coefficient (based on two-sided *t* test) is <2e-16 in the true data (left) for common, selective, and nonessential genes. To assess the significance of the relationship between standard deviation within a gene and biological correlations with the CERES scores of another gene, one example of a permuted CERES dataset and its linear model is shown in the middle. In total, 163 permutations of the CERES scores and the *P* values from their respective linear models are shown in the histogram to the far right. Permuted *P* values rarely drop below 0.001 and are never below 10^−6^ while the real data had a *P* value of <2e-16. The more context-specific a gene is, the more likely it is to be highly correlated to a second gene. Correlation with a second gene is an indicator that CERES score variation has biological meaning. **c** Different variables can be used to predict cell type specific genes. **d** The distribution of the number of features per data source. **e** gprofiler2 plots reveal the landscape of enriched biological processes in highly context-specific genes. The *P* values are based on hypergeometric tests with multiple testing corrections using the g:SCS method. **f** Comparisons in model scores (R2s) between the multivariate versus univariate models across different essentiality classes (*n* = 3648, 164, 22 for selective essential, common essential, common nonessential, respectively). Boxplots are broken down by gene classification, score is based on performance during cross-validation. Box shows the quartiles, whiskers indicate the maximum/minimum non-outlier observations, dots indicate outliers falling outside of 1.5 times interquartile range, and center is the median. **g** After cross-validation and validation of test set predictive power, models for each context-specific gene are plotted as individual points. 1 data point is 1 gene. Multivariate models (*x* axis) are more predictive than univariate models (*y* axis), across all gene essentiality classes. **h** Bar plot of Pearson correlations between model predictions and actual CERES scores, compared between the final modeling approach and a baseline model that simply predicts CERES score values by retrieving the CERES score from the closest cell line (by genomic profile similarities that are measured with Cellector). Source data are provided as a Source Data file.

In considering how to model cell-type-specific phenotypes, we consider the possibility that genes could follow two distinct models of cell specificity. For example, in one widely used model, CERES scores are thought to belong to two distinct probability distributions that are simply “essential” or “not essential” for growth in an individual cell line. This first model suggests that individual cell lines can be assigned one of these two classes for any gene. However, CERES scores in common essential genes can harbor large variation across cell lines^23–26^. Moreover, some genes that are labeled “common essential”, “selective essential”, and “non-essential” have large ranges of continuous variation in CERES scores that appear to be biologically meaningful (Fig. 1a, b). Thus, an alternative explanation is that CERES scores are continuous outputs of a single-cell type-specific function across cell lines that represent the degree to which gene loss-of-function changes cell growth rates^27^.

To be agnostic to these two models, we examined standard deviation and range filters across the CERES scores for all genes in all cell lines. This allowed us to identify the genes with the most context specificity across cell lines (i.e., increasing standard deviation and range). Interestingly, these genes tended to have continuously correlated non-paralogous genes in the dataset (Fig. 1b). This relationship was not simply a function of increased variation because permutation tests failed to observe similar trends in resampled data. Thus, we decided that a regression-based approach to cell-type specificity is reasonable because many highly context-specific genes have continuous CERES values that appear to be biologically meaningful. We visualized distributions and identified 583 genes out of 18,333 total genes using a cutoff (see “Methods”) that identified the largest standard deviations and the most expansive ranges of CERES scores across all cell lines. (Supplementary Fig. 1a). These highly context-specific genes were enriched for functions in the cell cycle, metabolic processes, and membrane-bound organelles (Fig. 1e and Supplementary Data 1) and they form the basis for our initial analysis of cell-type specificity.

There are multiple potentially predictive data types (and millions of data points) assayed in the DepMap project that could be used to infer the origins of context-specific loss-of-function phenotypes (Fig. 1c, d). All of the datasets (mutation, RNA-seq, CNV, and lineage) that we examined from the CCLE contained a large dynamic range of measurements (Supplementary Figs. 1b and  2). This suggested that variation in any/all the data types could be useful for making context-specific predictions of CRISPR loss-of-function phenotypes and inferring predictive features of interest.

Traditional definitions of synthetic lethality in cell lines examine univariate genetic predictors that include copy number and mutation context. Beyond classic definitions of synthetic lethality, lineage and transcriptional data have also been used to mine large genetic datasets. Because we aim to understand the nature of context specificity and how predictable it is, we took a fundamentally different approach from recent impressive work that ranked potential univariate biomarkers and drug target pairs^4^. Compared to univariate predictors, our first question was whether multivariate machine-learning models can explain larger amounts of context specificity. We also dramatically broadened the scope of our inquiry to include applications in biotechnology by allowing a CERES score in one gene to be predicted by other CERES scores across the genome. We did this because we suspected that the collective probing of the loss-of-function dynamics of the entire genome through CRISPR could provide information on the state of the genetic network that is fundamentally different than baseline OMICS measurements in unperturbed cells.

With millions of measurements, we needed to build an extensive feature selection and model validation pipeline for our machine-learning models to minimize overfitting while ensuring robustness and predictive power (Supplementary Fig. 3). Comparing across multiple machine-learning methods, we found that an approach that employed iterative-feature selection combined with random forest regression was robust and superior to other approaches (Supplementary Figs. 4 and 5). We validated our models with additional test sets (from later Broad data releases) and we observed comparable results (Supplementary Fig. 5a–c).

With this high-quality pipeline, we built thousands of machine-learning models across millions of measurements. We found that multivariate models virtually always outperform univariate models when predicting diverse cell-line-specific phenotypes across our set of highly context-specific genes (Fig. 1f, g). This suggests that multivariate context critically improves predictions of cell-type specificity, and that classic synthetic lethality as a univariate paradigm to interpret context specificity can be greatly improved upon. We next aimed to compare our findings versus a null model of cell-type specificity. We reasoned that a nearest-neighbors approach, whereby CERES scores in a test set cell line are predicted by the nearest cell-line neighbors in the OMIC data (i.e., cells that are most similar by mutation status/transcriptome/copy number via CELLigner). We observed that multivariate models using our machine-learning pipeline dramatically outperform this null model (Fig. 1h), as well as other machine-learning approaches (Supplementary Fig. 4). However, are our multivariate models better because they add further genetic context to key synthetic lethal mutations? Or is the multivariate prediction accuracy due to something else?

### CRISPR phenotypes predict cell-line-specific responses to gene loss

**Fig. 2:**
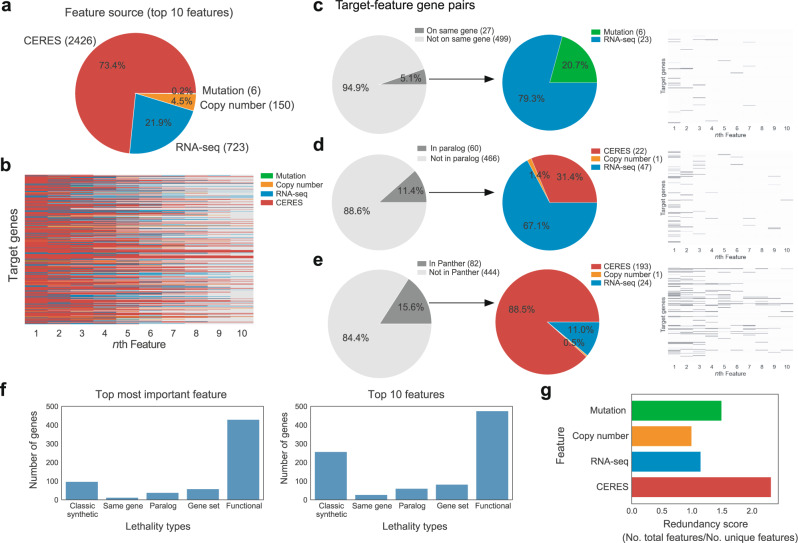
CRISPR CERES scores are the most prominent predictive features in multivariate models. **a** The distribution of the top ten features per data source from the multivariate models in Fig. 1 are aggregated across all models. CERES scores are highly enriched as features in these multivariate models. **b** A heatmap view of all target genes for *n*th feature, colored by the data source. Gray indicates there were less than ten predictive features for the target gene. **c**–**e** The relationship between the top ten features for every cell-type-specific gene and whether they are from the same gene (**c**), paralogs (**d**), or in network gene set Panther (**e**), as visualized in the gray pie charts and heatmaps. Of the feature-target gene pairs within each group (dark gray), they are further broken by data source (colored pie charts). **f** Examining the top feature in every model, we classified which model of cell-type specificity that feature belonged to. **g** Redundancy scores are measured as the ratio of the total number of features to the total number of unique features, measured per data source (CERES, RNA-seq, mutation, copy number, lineage). Source data are provided as a Source Data file.

To investigate why our multivariate machine-learning models make better predictions than univariate models, we first examined the types of data that contribute to highly predictive models of context specificity. A plurality of input features (40.0%) were RNA-seq transcripts in the input dataset, but the top ten features in our predictive multivariate models were overwhelmingly composed of CERES scores (73.4%) (*P* value <0.001) (Fig. 2a, b and Supplementary Figs. 6a and  7). This suggested to us that perturbations of the genetic network (as provoked by Cas9, and measured by CERES scores) were better at predicting cell-type-specific phenotypes than conventional “omic” measurements from unperturbed cells.

To quantify the degree of these differences and to understand them, we examined our multivariate models of context-specific loss-of-function using existing biological models of context specificity. These alternate biological models include classic synthetic lethality (the genotype of cell x predicts sensitivity to LOF), dosage lethality (the expression level of the “same gene” predicts its own sensitivity to knockout and is referred to as the “same gene” in Fig. 2), paralog lethality (the loss of a compensating paralog confers sensitivity to a related gene), lineage lethality (where cell type or developmental context predict genetic sensitivity) and network context (where neighboring genetic nodes in a network diagram, or sets of genes, are connected using another database that adds signal to noise in context-specific predictions, referred to as “gene set” in Fig. 2)^4,18,28–30^. These were compared to a “functional” model (Fig. 2) that used CERES scores as predictive variables. Among the top ten features in our multivariate models, all of these existing models of context specificity were observed (Fig. 2c–f). This is consistent with exciting recent papers where similar approaches have featured prominently^4,28,30^. Moreover, it re-emphasizes the importance of these relationships in a only select group of context-specific genes. We found >4 times as many context-specific CRISPR loss-of-function phenotypes were accurately predicted by the CERES scores of another gene in the genome (Fig. 2f).

Therefore, prior paradigms do not capture as many context-specific relationships as functional genetic CRISPR features. Importantly, this also represents a distinct model of how context specificity can be generated. In classic synthetic lethality, cell-type specificity is predicted by mutations that are unique to a subset of cells. Synthetic lethal relationships suggest that single “private” mutations cause a genetic network to function in two qualitatively different ways in two different cell types. We refer to these as “private” models, because a subset of cell lines harbors a “private” genetic mutation that predicts a unique response to CRISPR mediated loss-of-function. In contrast, our models utilize measurements of the function of predominantly wild-type genes that are consistently expressed across all cell lines, therefore we refer to our models as “public” models because the topology is “public” to all cell lines, and quantitative differences in pathway utilization account for context specificity. Just 6.9% of the genes with significant CERES scores have any alterations in any of the omic data in any cell line. Thus, our “public” models are measuring the function of wild-type genes that are utilized differentially. This is a different way to explain context specificity. It implies that many of the common rules for the utilization of wild-type genes are used across all cell lines and that cell-line specificity can be driven by the degree of common pathway utilization. Importantly, this is true in genes that are labeled as “selective essential” and “common essential”.

To understand this common genetic architecture in more detail, we also sought to understand the topology of the combined set of all multivariate models. In other words, we asked how many predictive features are shared between models. If multiple models share the same CRISPR features, it would suggest that some genes might be especially good at predicting context specificity, and it could give us insights into the common genetic architecture of human cell lines. It would also suggest that some screening information might be redundant. We divided the total number of predictive relationships by the number of unique predictors to get the average number of predictive relationships per feature. CRISPR features had an average of two relationship/feature, meaning that unlike mutation predictors, many CRISPR gene features were shared across multiple models and that redundancy is possible (Fig. 2g).

### Densely cross-predictive CRISPR networks define a common genetic architecture in mammalian cells

**Fig. 3:**
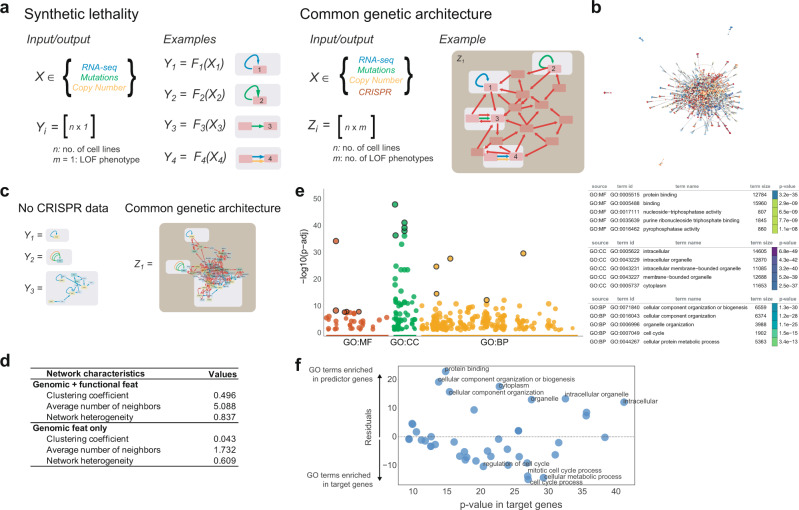
A common genetic architecture in mammalian cell lines. **a** Schematic illustrating difference between classic synthetic lethality and our *common genetic architecture*. Synthetic lethality consists of many individual *Y*_*i*_ functions. These functions are cell-type-specific models with single features. Our proposed common genetic architecture is hypothesized to connect these “private” functions with shared CERES features. A common genetic architecture has many redundant edges, and more interconnected nodes. More nodes suggest that more cell-type-specific phenotypes are predictable, and more edges suggest redundancy. **b** A network built from the aggregation of all multivariate models. Genes are represented as nodes and feature-target gene relations as edges. Colors represent distinct subnetwork communities that were identified by the Louvain method. **c** Network communities with (right) and without (left) nodes/edges involving functional CRISPR features for a single Louvain community, and a comparison with our hypothesis from (**a**). Edges are colored based upon the data source; and nodes are colored based on the model score (of top ten feature model) of the corresponding gene as target. **d** To quantitate the visual similarity between our hypothesis in (**a**) and the data in (**c**) across all Louvain communities, we examined the differences in the clustering coefficient, the average number of neighbors, and the network heterogeneity. **e** gprofiler2 plots examine the enrichment of functional categories. **f** Residual plot identifies GO terms that are more (residuals of −log10 *P* values >10) or less (residuals of -log10 *P* values < −10) enriched in predictor genes than in target genes. Dots represent shared GO terms among the 100 most significant terms in target and predictor gprofiler2 analysis result. The *p-*values from gprofiler2 for (**e**) and (**f**) are based on hypergeometric tests with multiple testing corrections using the g:SCS method. Source data are provided as a Source Data file.

Because of the dichotomy between the single “private” genetic defects that predict context specificity for single genes, and the “public” features in our models, we provide a schematic that describes our hypothesis to account for these differences. In cancer models of synthetic lethality, a variety of possible models exist. These distinct relationships form distinct “private” functions *f*_*i*_*(x)* that predict cell-type-specific differences in CERES scores *Y*_*i*_ (Fig. 3a, left). The predictive features in these functions are rarely shared between models (Fig. 2g). However, in a common genetic architecture, these “private” functions exist in the background of a densely predictive common genetic architecture that ties many individual *Y*_*i*_ predictions together, and many CERES features are shared between models (Fig. 3a, right). This hypothesizes a different network structure. We asked whether this schematic was consistent with our models of the DepMap data by using the union of all models to build networks where genes are nodes, and predictors of cell-type-specific phenotypes are edges (Fig. 3b). When we compared the networks with and without CRISPR CERES scores, we observed a striking dichotomy (Fig. 3c, d and Supplementary Fig. S8) that matched our hypothesis from Fig. 3a. CRISPR CERES predictions connect many “private” models into larger interconnected subnetworks (Fig. 3c, d and Supplementary Fig. S8), this dramatically increases the number of nodes (genes) that exist in the network (i.e., It explains more context specificity), and these nodes have a higher mean number of neighbors 5.1 vs 1.7 (*P* value = 2.0e-24), and a higher clustering coefficient 0.5 vs 0.04 (*P* value = 1.7e-40) (Fig. 3d). These highly connected and densely clustered subnetworks identify the fabric of genetic interactions that are interwoven to collectively determine cell specificity across cell lines. To understand the biological nature of this network, we examined the difference between the biology of cell-specific genes versus the common genetic architecture of predictive features that connect highly cell-type-specific genes using gprofiler2. Whereas cell-type-specific genes govern the cell cycle or metabolism (i.e., core cellular functions), the underlying biology of the genes that predict cell specificity was enriched for genes that coordinate biology across the cell’s various organelles and protein complexes (Fig. 3e and Supplementary Data 2). This increase in terms referring to the cellular organization, and protein–protein binding in the genetic architecture genes can be visualized in a residuals plot in Fig. 3f, where the terms that are the most uniquely significant in either gene set are highlighted by *P* value residual. Thus, the “common genetic architecture” can be rationalized by the cell biological function of genes that coordinate the activity of individual organelles and proteins across the cell.

**Fig. 4:**
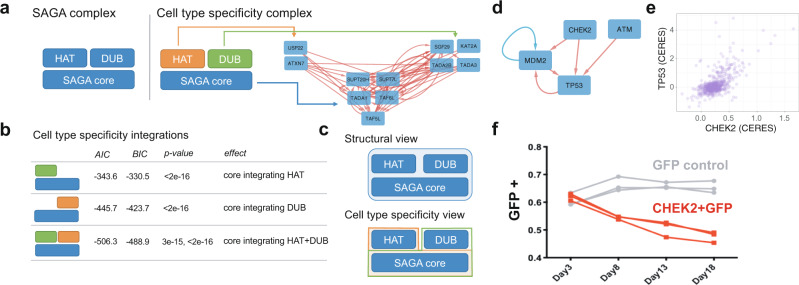
Two vignettes on cell-type-specific prediction networks. **a** A simplistic diagram of key functional modules in the SAGA complex includes a Histone Acetyl Transferase (HAT) module, a De-Ubiquitinating (DUB) module and core complex members (SAGA core) that are thought to be structural. The subnetwork to the right was extracted from a Louvain community containing the SAGA complex and its associated enzymatic modules. The edges are predictive relationships of CERES scores in our final models. When comparing the density of the edges between functional modules, the sparsity of the red edges between HAT and DUB modules is visually striking (comparing green to orange vs. green to blue and orange to blue). **b** A simple multivariate linear regression model (for interpretability) was used to rationalize the network structure. The aggregate measures of cell-type-specific phenotypes for each functional model were created by averaging the CERES scores of individual genes in each module from (**a**). HAT or DUB, as well as HAT and DUB cell-type-specific features, were found to be independent predictors of the cell-type-specific phenotype of the SAGA core complex (measured by the Akaike information criterion (AIC), Baye’s information criterion (BIC), and *P* value of model coefficients based on two-sided *t* tests). **c** A diagram of the difference between the static, structural, view of the SAGA complex that suggests an integrated molecular machine that is built from its constituent modules, and a cell-type specificity view of the SAGA complex that suggests plasticity in module requirements across cell lines is genetically integrated by the SAGA core. **d** *CHEK2* and *TP53* interactions in our functional networks are presented from their Louvain community. Red arrow are CERES prediction relationships and the blue arrow is an RNA-seq feature. **e** CERES scores for *TP53* and *CHEK2* across all cell lines in the Broad institute’s dependency map data (19Q3). **f** To demonstrate the role of *CHEK2* in the absence of DNA damage in already transformed cell lines, we performed an “add back” experiment where we infected *CHEK2* mutant Colo678 cells with a functional copy of *CHEK2* marked with GFP. Partially transduced cells can be used to measure fitness changes via a GFP competition experiment. Partially infected pools are monitored for the GFP + proportion of cells *(n* = 3 replicates). Depletion occurs when the fitness of the infected cell decreases. The control is GFP alone. Source data are provided as a Source Data file.

To get a closer look at how predictive features coordinate cell-type-specific genes, we examined a specific network in greater detail. We chose a network that harbored the SAGA complex (Fig. 4a). SAGA is involved in co-transcriptional activation from yeast to metazoans. It acetylates and deubiquitylates histones in promoter regions to co-activate transcription by RNA polymerase II^31,32^. These activities are thought to act in a concerted fashion and a recent cryo-EM structure links the human SAGA complex’s canonical acetyltransferase and deubiquitinylation activity through a core assembly of SUPT20H, TAF6L, TAF5L, TADA1, and SUPT7L (among others)^33^ (Fig. 4a). The core is thought to physically link both enzymatic functions to promote robust transcriptional co-activation. Our models of cell-specific phenotypes easily predicted which cell lines were sensitive to the depletion of core SAGA complex genes by using the CERES scores of other SAGA core complex members (Fig. 4a). Thus, the structure of the SAGA core complex has similarities to our functional networks of cell-type specificity.

Interestingly, while the core SAGA genes were densely cross-predictive of each other, the view of the complex, in light of our cell-type specificity networks, has a different interpretation than a cryo-EM structure. The enzymatic genes USP22 and KAT2A and others in these sub-modules were not cross-predictive of each other (Fig. 4a). A resampling analysis suggested that the lack of cross predictions between enzymatic components was statistically significant (*P* < 0.05). The simplest null hypothesis for cell-type specificity in a complex is that an entire complex is a single machine and that more or less of the entire machine is required in some cell lines versus others. If this is the case for the SAGA complex then both enzymes would be expected to be tightly linked to SAGA core complex phenotypes, and to each other.

Here, the protein scaffold (the SAGA core) that physically associates the enzymatic functions (USP22 (DUB) and KAT2A (HAT)) appears to offer a more surprising role in cell-type specificity. The scaffold serves to integrate incoherent variation in the cell-type specificity of enzymatic activities and it does not form a single molecular machine. This can be seen in a simple regression model using USP22 and KAT2A as independent predictors of cell-type-specific SAGA complex phenotypes. This model results in significant, independent, and additive contributions of both enzyme subunits to the prediction of core SAGA essentiality (see Fig. 4b, c; AIC, BIC, and *p-*values of variables). Thus, SAGA cell-specific phenotypes are computed using a weighted sum of the USP22 and KAT2A dependencies, suggesting that the core complexes’ context-specific role is to coordinate incoherent dependence of its substituent enzymatic activities.

Subnetworks also highlight coherent biology (Supplementary Fig. 8a–c), Supplementary Fig. 8b shows a Cyclin-CDK-regulatory network that is entirely composed of genes that drive the cell cycle. Supplementary Fig. 8c highlights mitochondrial respiration and the electron transport chain. To get deeper biological insights, we also decided to look carefully at specific networks surrounding the TP53 gene in Fig. 4d. While TP53 is part of many pathways, two well-studied pathways are ATM- > CHEK2- > TP53 and ATR- > CHEK1- > TP53. Both CHEK1 and CHEK2 can directly phosphorylate TP53^34,35^, but CHEK1 and CHEK2 are typically studied following the application of external DNA damage, not during unstressed growth^36^. While these pathways appear straightforward in reviews^36^, the genetic evidence regarding the cell-type specificity of CHEK2 and P53 loss of function adds further confusion because MEFs grown from knockout mice suggest that ATM and P53 but not CHEK2 are required for cell cycle arrest following irradiation^37^. Moreover, in some cancer cell lines, CHEK2 affects cell viability following DNA damage in a TP53 independent manner^38–40^. Finally, while CHEK1 is likely a common essential gene in humans, TP53 is not^23^. Thus, the cell-type-specific genetic pathways regulating the cell-type-specific function of CHEK1 and 2 for normal growth are unclear, especially in the absence of external DNA damage and in fully transformed cancer cell lines.

To investigate this, we looked for edges between CHEK1 or CHEK2 and TP53 in our cell-type specificity models. We found that CHEK2, but not CHEK1 is a predictor of TP53 cell-type-specific KO phenotypes across all cell lines (Fig. 4d). A closer inspection of the data suggests that this relationship exists because sgRNAs that cause the loss of CHEK2 or the loss of TP53 enrich in the same subset of cell lines (Fig. 4d, right). This result was somewhat surprising to us because CHEK2 is not widely known to regulate the cellular fitness of already transformed cells in the absence of DNA damage and we could not find a clear precedent for it in the literature. It is formally possible that CRISPR-induced DNA damage is occurring and that CRISPR-induced double-strand breaks artificially cause cell lines with CHEK2 loss to enrich the screening data. An orthogonal experiment would be to show the opposite effect: i.e., that the re-addition of functional CHEK2 in a CHEK2 loss-of-function cell line can cause a growth defect. While CHEK2 loss-of-function mutations are rare in human cancer cell lines, we identified a cell line, Colo678, that harbored biallelic loss-of-function mutations in CHEK2. Re-expression of a functional WT copy of CHEK2 caused a growth defect in the absence of DNA damage, while expression of a GFP control did not. In prior literature, it has been suggested that the re-addition of TP53 to TP53-deficient cell lines is a potential cancer gene therapy strategy^41^. Our data suggest that a similar strategy of re-expressing CHEK2 in CHEK2-deficient cell lines is possible.

However, in spite of these intriguing biological vignettes, we believe our most important hypothesis is that not all genes in a subnetwork would need to be measured in order to predict the CERES scores across an entire subnetwork, and if successful, this would be as transformative for CRISPR screens as other recent work in transcriptomics^22^.

### Identification of a common genetic architecture leads to the lossy compression of CRISPR libraries

Measuring sgRNA enrichment or depletion in pooled screens informs gene function by identifying when genes are required for viability^23,24,42^. However, these libraries ‌can be‌ ‌too large and cumbersome‌ ‌for many types of biological studies. This is because CRISPR libraries require large amounts of coverage (4–10 sgRNAs/gene, 500 cells/sgRNA, and 500 reads/sgRNA) to achieve high-quality measurements^43–45^. A “simple” genome-wide dropout screen in a mammalian cell line can require 40 to >100 million cells and a similar number of sequencing reads to get good measurements on ~20,000 genes. Thus, an approach that decreases the measurement requirements at the genome scale would be useful for mammalian cell biology and genetics. This reduction creates a natural analogy to image compression. “CRISPR predicts CRISPR” models might be able to compress CRISPR experiments instead of their data. We aim to identify reduced sets of CRISPR constructs that could be screened at smaller scales to predict the loss-of-function effects of unmeasured genes at the genome scale.

**Fig. 5:**
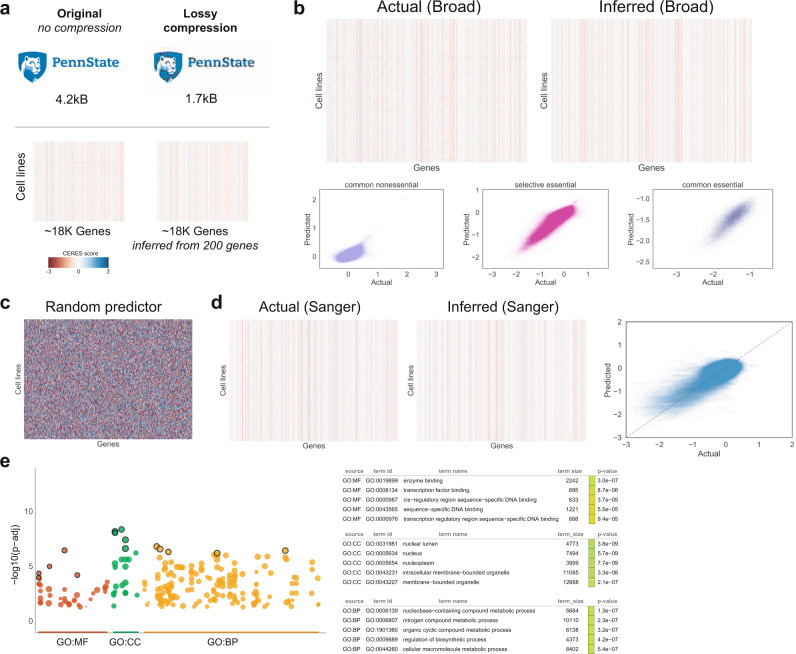
Lossy genomic compression. **a** Schematic of lossy compression in terms of image compression using JPEG (top) and similarity to genomic loss-of-function compression. **b** Genome-wide comparison between actual and inferred CERES scores on a held-out test set, shown as heatmaps and scatter plots per gene essentiality classes. Models built based on only 200 lossy genes with their CERES scores as features. **c** The same test set as in (**b**) but was inferred based on a random predictor. **d** Genome-wide comparison between actual and inferred CERES scores on the independent test set data from the Sanger Institute. Multivariate models were trained based on Broad data from 19Q3 and used to predict independent cell lines and an independent library. **e** gprofiler2 plots identify functional enrichments in lossy compression sets. The *P* values are based on hypergeometric tests with multiple testing corrections using the g:SCS method. Source data are provided as a Source Data file.

Image compression can be lossless or lossy. Lossless compression achieves modest reductions in file sizes but loses no information. Lossy compression can achieve orders of magnitude reductions in file size (Fig. 5a), but there are tunable reductions in image quality^46^. For our purposes, a dramatic reduction in library size with some information loss is preferable to lossless compression and a modest reduction in library sizes. This is because a fundamental barrier to the scalability of modern human functional genomics is library size^44^. Achieving less than an order of magnitude reduction in library size will be useful, but it will fail to dramatically enhance experimental tractability in many experimental contexts. For instance, even a genome-wide library of perfect sgRNAs cannot decrease CRISPR library sizes to below ~20,000 unique constructs. Eliminating the need to measure redundant genes (and not redundant sgRNAs) is the only approach that has the potential to create libraries that are orders of magnitude smaller.

Inspired by lossy compression and previous landmark analyses in transcriptomics^22^, we hypothesized that the existence of a common genetic architecture could enable the tunable lossy compression of the genes that are targeted in CRISPR libraries. Here, compression does not refer to the in silico data storage requirements, but to the in vitro number of genes that must be measured in a pooled screen to achieve a genome-scale “portrait” of gene function (Fig. 5a).

To examine the possibility for lossy compression we decided to start with the entire genome, and not simply focus on the genes that were highly cell-type-specific. Our aim was to identify a highly informative set of genes, for whom experimental measurement can accurately predict unmeasured CERES at a genomic scale. Using a multi-step machine-learning approach, we identified 25, 100, 200, and 300 gene sets whose CRISPR CERES scores were highly correlated with other genes in the 19Q3 Broad DepMap data. These potential compression sets were enriched for genes that are involved in enzyme binding, the nucleus, biosynthesis, and transcription factor activity (Fig. 5e and Supplementary Data 3). We then examined whether these small gene sets harbored predictive power across the genome. With models trained on 488 cell-line screens from the 19Q3 Broad data and then tested on 87 independent cell lines screened in 19Q4, we found that the predictive performance of lossy compression genes in a validation set began to saturate at 200 genes (Supplementary Fig. 9a). Across the genome, predictions from a lossy 200 set closely resembled the true test set data (Pearson’s *r* = 0.92) (Fig. 5b and Supplementary Fig. S9b–d). Importantly, the similarity between predicted and measured CERES scores existed across common essential, selective essential, and nonessential genes. The predictions were dramatically better than models built from a random set of 200 genes (Pearson’s *r* = 0.0) (Fig. 5c). To build intuition, two representative examples of two gene-specific models are available in Supplementary Fig. 9d and all 18,333 models can be reproducibly generated from source on GitHub. Importantly, some common essential and nonessential genes were predicted to have roughly the same phenotype in all cell lines. This is consistent with the interpretation that some essential genes are interpreted in a binary fashion by the cell.

Recent work comparing the Broad dataset with the Sanger dataset has identified high cross-dataset correlations and key differences for many shared genes^14^. These projects had differences in screen duration, media composition, and sgRNA identity. As an extraordinarily stringent test of our lossy 200 set, we examined the predictive performance of our lossy 200 gene set that was built on only Broad 19Q3 data in an independent test set from the Sanger Institute (Fig. 5d). This Sanger institute data used different cell lines, a different library, different timepoints, and different media conditions. Consistent with a lossy approach, our lossy 200 set reproduced a genome-scale portrait of CRISPR loss of function in the Sanger institute data that resembled performance in the DepMap test data (Pearson’s *r* = 0.78). This is despite the Sanger institute’s use of sgRNAs, and screening conditions that our lossy 200 gene set was not trained upon. This clearly suggests that small sets of 200 genes can provide reproducible genome-scale information.

To probe this further, we examined predictions outside the context of simple growth. To et al. performed multiple sgRNA screens in the presence of a variety of small-molecule perturbations^47^. We reprocessed the data of To et al. using the CERES pipeline. Then we computationally extracted the L200 and performed predictions on the phenotypes of the rest of the genes in the small-molecule datasets. Correlations between measurements and predictions were relatively high across all seven screens (Pearson’s *r* = 0.72, Supplementary Fig. 10). Moreover, we also reasoned that screen hits should be similar between measurements and predictions. To test this, we examined the top 500 hits in each chemical screen. In this “hit list”, 70% of the top 500 hits were in common between measurements and predictions across all seven screens (Supplementary Fig. 10).

### A series of lossy compression screens accurately predict loss-of-function phenotypes

**Fig. 6:**
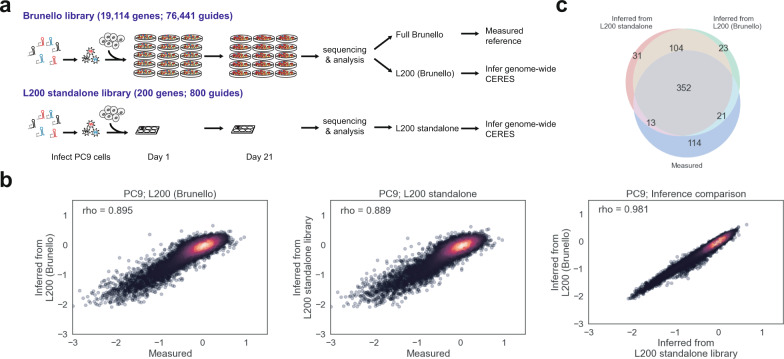
Direct experimental validation of lossy compression sets. **a** Two separate pooled screens were performed in a cell line (PC9) that was not included in model training and validation. Experiment 1 was the full Brunello library. A 21-day dropout experiment was performed in PC9 cells. Measurements on 18,114 genes were direct and form the gold standard. The L200 can be computationally extracted from the full screen and compared to these gold-standard measurements. A new L200 standalone library of 800 guides targeting 200 genes was cloned. This library can be used to perform a small-scale lossy compression experiment. The data can then be compared to the gold standard. **b** Correlations of inferred vs measured CERES scores for both screens in (**a**) and a comparison of the predictions between the standalone sets and the computationally extracted L200 set in the Brunello library. **c** A Venn diagram describes the overlap in “Hits” in the 500 most differentially required genes for growth in PC9 cells. Both of the lossy compression screens from (**a**) and the gold-standard (measured) data are compared. Source data are provided as a Source Data file.

Lossy compression is an exciting prediction. To test it experimentally, we focused on a cell line that was left out of the original analysis, PC9 cells. We ordered a third library, the Brunello library, which is the modern alternative to the Avana library and it shares only ~10% of the Avana guide sequences across more than 19,000 genes. We did two different experiments. The first was a pooled screen across the entire Brunello library. We then computationally extracted the L200 values and used them to predict all the measured values in the library (Fig. 6a). In parallel, we also cloned a standalone Lossy 200 library and we examined the predictions of the L200 set when the screen was performed in a 6-well plate. Both the standalone Lossy 200 set and the Lossy 200 set that had been computationally extracted from the full Brunello library were able to predict phenotypes across most of the Brunello library (Pearson’s *r* = 0.895 and 0.889, and the predictions between the approaches were remarkably similar, Pearson’s *r* = 0.981). In addition to correlations, we also examined “hit calls” in Fig. 6c because we know that functional genomics screens are often qualitatively interpreted as the top *N* hits in a screen. This Analysis showed that roughly 369/500 of the top hits measured in the full Brunello library in PC9 cells could be predicted from measurements of 200 genes.

## Discussion

Different cell types behave differently. This has been a fundamental question of genetics and cell biology since the first cells were cultured. It has guided classic work by Hayflick and Eagle^11,48^, as well as modern work in systems biology^49^. Recent systematic efforts to perform genome-scale CRISPR screens across mammalian cell lines are a significant new tool to understand this challenging question. This is due to the exceptionally detailed and comprehensive genomic information that now exists in CRISPR screened mammalian cell lines across two independent institutes^4,12,13^. Our work represents the largest systematic effort to use these datasets to understand the basic biological origins of cell-type specificity. Understanding this question advances basic cell biology and genetics. Previous work has focused on ranking translational hypotheses and comparing dataset measurement quality^4,14^. Surprisingly, well-described and clinically important phenomena like synthetic lethality can make strong models, but they are relatively rare explanations for context specificity across all cell lines/genes. We refer to these as “private” models, where a specific event in a subset of cell lines predicts a unique response to CRISPR mediated loss-of-function. Thus, “private” genetic mutations constitute a comparatively minor explanation for cell-type-specific phenotypes.

Instead of “private” models, the collective wisdom of all CRISPR loss-of-function perturbations across multiple genes constitutes a different model of cell-type specificity where the elements of genetic logic are shared across cell lines and they are linked by predictions that are enriched for genes that connect and coordinate diverse biological processes. Cell-type specificity is produced by the differential utilization of these connecting and coordinating genes across all cells. These models are based upon the information encoded in the response to stimuli in widely expressed genes that are not mutated. They can also be collectively assembled into a common genetic map that details the network architecture of cell-type-specific phenotypes across cells. Interestingly this common map resembles a decade-old concept in the signal transduction literature called “common effector processing” ^49^. Common effector processing describes cell-specific differences in caspase activation as a function of signal integration across a small set of widely expressed kinases and not the differential utilization of cell-type restricted kinase pathways. Thus, our genetic findings converge with classic high-profile work in post translational modification networks. This convergence points to a broader theme in explanations of context specificity that transcend a single-data type or phenotypic focus. This theme is that while discrete and qualitative differences between cells can drive cell-type-specific behaviors, it is often the quantitative degree to which ubiquitous pathways are utilized that determines context specificity. This is a continuous model of cell specificity in public genes, as opposed to the discrete model of synthetic lethality in “private” contexts. Our systematic analysis across the Broad and Sanger datasets lends support to this model of context specificity at a scale that dramatically exceeds prior work.

In network biology, previous work has focused on the most connected nodes. So-called “Network Hubs” are often enriched in functional phenotypes and regulate many genes^50^. In our study, the most connected hubs contain the most redundant features in our predictive models. This dichotomous interpretation of hubs (critical vs dispensable) is counterintuitive in network science because most work has identified network hubs as important. The highly connected genes in our common genetic architecture drive the insight that the lossy compression of CRISPR libraries is possible and it helps us understand which measurements we might be able to eliminate instead of the measurements that we want to keep.

While large screens of pools of guides targeting single genes in well-behaved cancer cell lines are easy to perform at the genome-wide scale, many contexts exist where the experimental coverage requirements of genome-wide libraries are logistically challenging, if not impossible. Lossy compression changes the experimental calculus by allowing a subset of a genetic library to predict unmeasured gene phenotypes. However, the largest caveat in our approach is that it is lossy, and therefore it has less information than a genome-wide library. This tradeoff is most important to consider in the set of genes for which zero significant phenotypes have been identified in any mammalian cell line in the Broad and Sanger data sets. Evidence in yeast and worms suggests that broader environmental and developmental contexts elicit more phenotypes across single gene knockouts^51,52^. Thus, the number of non-essentials decreases as more screening conditions are tested. Some completely novel phenotypes in a unique environment in a nonessential gene might be challenging to predict with our current lossy 200 compression set. However, new screens are being completed all the time that can help improve our lossy sets. At the time of our writing, nearly 1000 different mammalian cell lines have already been screened by the Broad and Sanger Institute. Only 1144 contexts were needed to identify a measurable phenotype in every yeast gene^52^. While the human genome will likely require more contexts to saturate phenotypic predictions, lossy sets may strongly benefit from approaches like transfer learning and few-shot learning as new data arises^53^. On the other side of this caveat, there is a clear limit to potential false negatives because many nonessential genes belong to paralogous gene families and contain genes that are likely to buffer each other’s effects upon single sgRNA knockout. Thus, many current nonessential genes may never have a measurable phenotype in any context with a single sgRNA^30^. Our current lossy 200 approach would be sufficient to predict the phenotypes in these genes in any context. This buffering provides a biologically plausible bound to potential false negatives.

Despite this caveat, lossy compression has enormous potential. Chemogenomic screens that kill cells can bottleneck libraries by tenfold at a 90% inhibition of cell viability. These bottlenecks in screens can raise coverage requirements for genome-scale screens to as many as 1 billion cells, a challenging bar. Beyond gene–drug interaction screens, pairwise genetic epistasis maps require *n*^*2*^*/2* unique constructs^44^. To make a genome-wide pairwise interaction map (even ignoring the challenge of cloning a pairwise library of 1e8 gene pairs), coverage requirements of 3 sgRNAs per gene, 500 cells/sgRNA, and 500 reads/sgRNA create an impossibly large screening challenge that requires a population greater than 10^10^ cells. Engineering cell lines to perform better for biotechnology applications could benefit from highly combinatorial genome engineering through CRISPR loss or gain of function. Testing all three-, four-, or five-way loss-of-function genotypes for enhanced performance in stirred-tank bioreactors is impossible without some form of library compression. Because thousands of unmeasured phenotypes can be predicted with hundreds of CRISPR measurements, lossy compression sets are positioned to expand the experimental landscape of possibilities in mammalian functional genomics by enabling genome-scale experiments that were previously impossible.

## Methods

### Datasets

Cancer Dependency Map datasets 19Q3 and 19Q4 were retrieved from DepMap portal^25^. This included the Broad and Sanger gene effects (CERES scores), CCLE expression, CCLE gene copy number, CCLE mutations, and lineage (from sample info file). The classifications of non-essentials and common essentials were also retrieved for the same release periods. The data used in this study are available in the Zenodo database under the DOI identifier (10.5281/zenodo.5721869).

### Data preprocessing

The data were pre-processed and later models were built using python. Several steps were performed to preprocess the raw data. Copy number missing value was replaced with zero. Cell lines with any missing CERES values were dropped. Mutations were grouped into damaging (with variant annotation labeled as damaging), hotspot nondamaging (with variant annotation labeled as not damaging and is either a COSMIC or TCGA hotspot), or other (with variant annotation labeled as other conserving/nonconserving).

Feature pruning was performed to remove noninformative variables. This included removal of features with only constant values and categorical features where if a category value was supported only by less than or equal to ten samples. Non-expressed genes (TPM < 1) were also removed. Data were normalized (*z* scored) for RNA-seq and copy number values.

### Feature selection and model building

The model building was based on an iterative-feature selection process. First, the dataset was constructed using CERES (excluding the target gene), RNA-seq, copy number, mutations, and lineage as features, with the goal of predicting the CERES of a target gene. The target genes list was a set of 583 genes with highly variable phenotypes based on CERES standard deviation >0.25 and range >0.6. For lossy gene set compression inference (described below), the target genes list was the entire genomic set of 18,333 genes. The data were split 85% and 15% into train and test sets, respectively. The full training dataset was fit using a random forest regressor with 1000 trees, a maximum depth of 15 per tree, minimum of 5 samples required per leaf node, and a maximum number of features as log2 of the total number of features. The top quartile of most important features was kept and used to refit a new random forest model. This process was repeated three times. The remaining feature set was further refined for significant features using the Boruta feature selection method^54^. The resulting features fit using random forest constituted the reduced model. For the purposes of analyses, the top ten most important features were selected and the resulting model constituted the top ten only features model. These were multivariate models (i.e., more than one feature genes were used for the prediction). In the case of comparison to univariate models, for each target gene, additional univariate models were built (one per feature gene) as random forest models.

In addition to this iterative-feature selection and Boruta selection with random forest, other modeling approaches were performed for comparison. This included linear regression, elastic net (with alpha of 0.1), and random forest on the full train dataset followed by taking the top quartile of important features for building the reduced model. The top ten most important features were then taken for follow-up analyses.

For the Nearest-Neighbors Null model, the Celligner-aligned cell-line UMAP positions were retrieved from the Cancer Dependency Map portal (https://depmap.org/portal/celligner). The Euclidean distance between every cell-line pair was calculated using the UMAP 2D positions. The predicted CERES score in a test cell line was one of its nearest cell-line neighbors. Pearson correlation was then calculated between the predicted and actual CERES scores.

### Model evaluation

The models were evaluated based on *R*^2^ values and were calculated for the full, reduced, and top ten only features models. While *R*^2^ values provide a sense of model fit, it does not describe model significance. To this end, a recall metric was also calculated to take into account the correlation between predicted versus actual CERES relative to a reference null distribution. More specifically, the recall was defined, similar to that in Subramanian et al.^22^, as the fraction of null Spearman correlation values that was lower than the Spearman correlation of the given model for the target gene of interest. The null correlation value was calculated as the Spearman correlation between the actual CERES of a randomly drawn gene and the predicted CERES of the target gene. This procedure was repeated 1000 times to generate the null distribution, per target gene. For the analysis set, we focused on predictable models with a recall >0.95 and *R*^2^ > 0.1 of the top ten only features models (resulting in 529 target genes); and with at least one univariate *R*^2^s (of the top ten univariate *R*^2^s) >0. With these filtering criteria, the initial 583 target genes were reduced to 526 highly predictive target genes. In the analyses of feature genes, we excluded low-quality feature genes where the univariate R2s were ≤0. In addition, a concordance score was calculated per model as the fraction of all predicted CERES values where the actual and predicted were both below or above −0.6 (a cutoff used for essentiality).

### Gene set analysis

Gene set enrichment on context-specific genes, common genetic architecture predictor genes, and lossy200 genes were performed using gprofiler2^55^ in *R* with default parameters and a *P* value significance threshold of 0.05. gprofiler2 *P* values of top terms in common genetic architecture predictor genes were regressed on *P* values of top terms in context-specific genes and residuals were visualized with seaborn *residplot*.

### Network analyses

Networks were derived from the model results by linking all related source and target genes using *networkx* in python. Communities were extracted from this network using the Louvain method. Network communities were visualized and network statistics were extracted using Cytoscape v3.7.2^56^. Power analysis was performed on nodes with undirected degree of at least 2. The number of nodes vs degree was fit to a power-law function Eq. (1).

1 $$y=a{x}^{b}$$

### Lossy gene sets

Lossy sets were derived as centroids in an iterative *k*-means-based tight-clustering procedure using the *tightClust* package in *R*^57^. The procedure was run to derive L25, L75, L100, L200, and L300 lossy sets, which were then used for the saturation analyses. For compression-based inference, the input dataset consisted of the CERES of these lossy gene sets, as opposed to all genomic features.

### Statistical analyses

The enrichment of contribution of CERES as features was statistically tested using Chi-squared test. The statistical significance for node clustering coefficient and the average number of neighbors between functional only and functional + genomic networks was assessed using two-sided *t* test. All statistical tests were performed in python.

### CHEK2 construct generation

CHEK2 cDNA was synthesized in-frame and linked to 3’ GFP (from pLVX-PGK-Puro-IRS-GFP) by a triglycine linker. The GFP-CHEK2 DNA fragment was then cloned into pLVX-IRES-Puro using recombination-based cloning.

### COLO678 CHEK2 competition experiment

The constructs were co-transfected into HEK293T cells using calcium phosphate with 3rd generation lentivirus packaging vectors. On the same day, COLO678 (DSMZ, ACC#194) were seeded at 0.6 M/ml, 1 ml per well in six-well plates. One day after transfection, the media were changed to RPMI to collect viruses. Twenty-four hours later, cells were infected with 1 ml of virus supernatant and 4 mg/mL polybrene. The growth rate monitoring was started three days after infection. The cells were trypsinized and the growth of successfully infected cells (GFP positive) was quantified with flow cytometry every 5 days.

### Lentiviral sgRNA library preparation

Human Brunello CRISPR knockout pooled library was a gift from David Root and John Doench (Addgene #73178). Lentivirus was generated by co-transfecting 20 10-cm plates of HEK293T cells with the pooled library and third-generation packaging vectors using Lipofectamine 2000. Virus was harvested 48- and 72-h post transfection. The viral supernatant was precipitated by adding 80 µg/mL polybrene and 80 µg/mL chondroitin sulfate, incubating at 37 °C for 20 min, and centrifuging 10 k *g*’s for 5 min. The resulting pellet was resuspended in 15 mL RPMI, aliquoted, and frozen at −80 °C.

### Brunello screen in PC9 Cells

The titer of the viral library was measured with a test infection of PC9 cells (ATCC CRL-11350) 12-well plates. After 20 h of exposure to virus, cells were removed from the virus and cultured in 1 µg/mL puromycin for 3 days. For the full-scale screen, infection conditions that yielded a 10% infection efficiency were chosen to achieve a post-selection MOI of ~1. To achieve 150-fold library cover after selection, 13 million cells were infected in 12-well plates at 250 k cells/well. After infection, cells were cultured in 1 µg/mL puromycin for 3 days, and the 10% infection efficiency was confirmed by comparing cell counts in wells with and without puromycin. After selection, cells were maintained in T175 flasks, with passage every 3 days to maintain a minimum of 500-fold library coverage (minimum of 38 million cells). Cells were harvested at day 21, and gDNA was extracted from a pellet of 35 million cells (~450-fold library coverage). The guide library was prepared using the Brunello sequencing protocol provided by Addgene. The library was sequenced on a HiSeq 3000 (single-read 50 bp).

### Standalone lossy 200 screen in PC9 cells

The standalone Lossy 200 screen in PC9 cells was performed similar to the full-scale Brunello screen. The subset of L200 gene guides from the Avana library (800 guides) were synthesized and cloned into lentiCRISPRv2 (Addgene #52961). Successful library coverage was confirmed with NGS. The L200 lentivirus was prepared in two 10-cm plates, similar to the Brunello virus, and titered to achieve 10% infection efficiency. Three million PC9 cells were infected in order to achieve ~225-fold library coverage per replicate following selection. Infected cells were maintained in 10-cm plates, passaging to a minimum of 400 k cells (500-fold library coverage). Cells were harvested at day 21, and gDNA was extracted from a pellet of 1 million cells (~1250-fold library coverage). The guide library was prepared and sequenced as in the Brunello screen.

### CERES score analysis in L200 standalone screen

Guide counts were determined from the sequencing data using the Broad Institute’s PoolQ shell script. Log-fold change in guide abundance was calculated relative to pDNA values. The log-fold changes were subjected to the QC metrics outlined in Dempster 2019^14^Gene essentiality was inferred using the R package *ceres*, and scores were scaled such that the median essential gene and nonessential gene (from the list developed by Hart et al.^23^) core was −1 and 0, respectively. For the standalone L200 screen, there was no overlap between the L200 gene set and the nonessential gene set. In this case, the depletion values were scaled such that the median essential gene score was −1, and the median conditionally essential gene effect matched that of the average conditionally essential gene scores across all cell lines in the Achilles dataset.

### Brunello PC9 and To et al. data analysis

The 19Q3 CERES scores were used as training data for model construction. For model predictions, the To et al.^47^ and Brunello PC9 L200 CERES scores were extracted and used to predict the other measured scores in Brunello PC9 and To et al*.*^47^ drug screen. For the standalone PC L200 screens, the standalone L200 values were used to predict the other measured values in Brunello PC9 data. Data preprocessing and feature selection of these external data were followed^47^.

### Reporting summary

Further information on research design is available in the Nature Research Reporting Summary linked to this article.

## Supplementary information

Supplementary Information

Description of Additional Supplementary Files

Supplementary Data 1

Supplementary Data 2

Supplementary Data 3

Reporting Summary

## Data Availability

All original data are publicly available on the DepMap portal (https://depmap.org/portal/download/) and upon download can be placed in the data folder as described in the documentation in our GitHub repository [https://github.com/pritchardlabatpsu/cga]. Source data are provided with this paper.

## Source data

Source Data

## Code availability

A fully documented git repository with all source codes and notebooks can be accessed at the Pritchard Lab at PSU GitHub repository [https://github.com/pritchardlabatpsu/cga]. The source codes for running the entire pipeline have been provided as a python package, with all library dependencies defined for complete reproducibility— including the virtual environment used. The repository also contains codes for the generation of all figures/tables and accompanying source data presented in this paper.
