# Supplementary material for: A pan-CRISPR analysis of mammalian cell specificity identifies ultra-compact sgRNA subsets for genome-scale experiments
Source: Nat Commun. 2022 Feb 2;13:625. doi: 10.1038/s41467-022-28045-w (PMC8810922; doi:10.1038/s41467-022-28045-w)
Supplement: Supplementary file 1 — Supplementary Information [file 41467_2022_28045_MOESM1_ESM.pdf]

A pan-CRISPR analysis of mammalian cell specificity identifies ultra-compact sgRNA subsets for genome-scale experiments

Zhao *et al.*

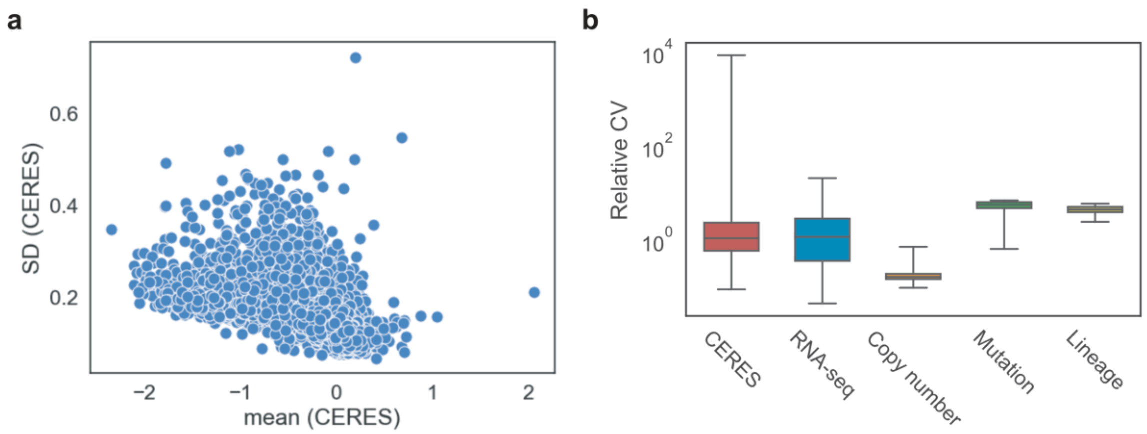

Supplementary Figure 1. Baseline statistics of DepMap datasets. (a) Scatter plot of standard deviation (SD) versus mean of CERES scores. (b) Relative coefficient of variation (CV) of each data source. Relative CV is calculated as the ratio of standard deviation to the absolute value of the mean. For this box plot, box shows the quartiles, whiskers indicate the maximum/minimum non-outlier observations, dots indicate outliers falling outside of 1.5 times interquartile range, and center is the median. Total n each for each source are CERES: 18333; RNA-seq: 36759; copy number: 27639; mutation: 9065; lineage: 18. Source data are provided as a Source Data file.

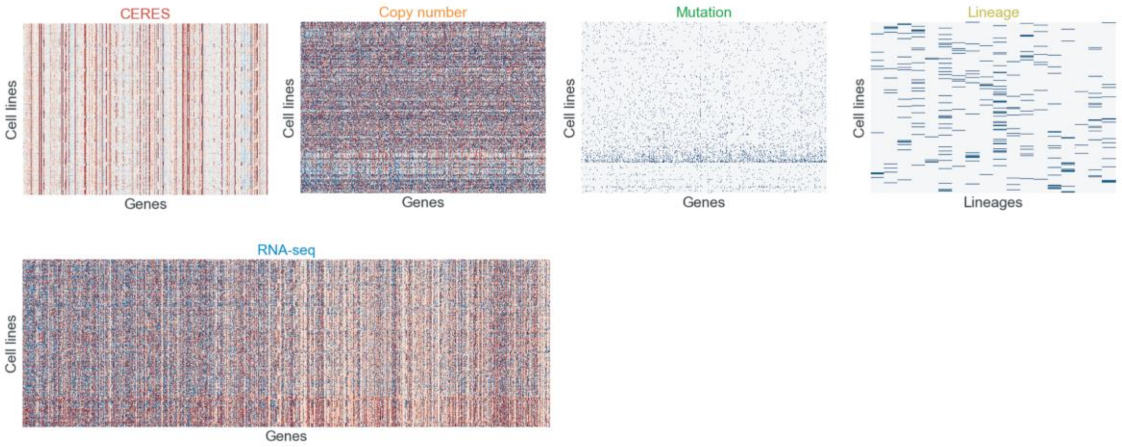

Supplementary Figure 2. Heatmaps of diverse datasets from DepMap. Heatmaps of datasets available for the prediction of context specificity from the 19Q3 DepMap CERES scores, copy number, RNA-seq, mutation, and lineage. The colors were scaled to be between -1 and 1.

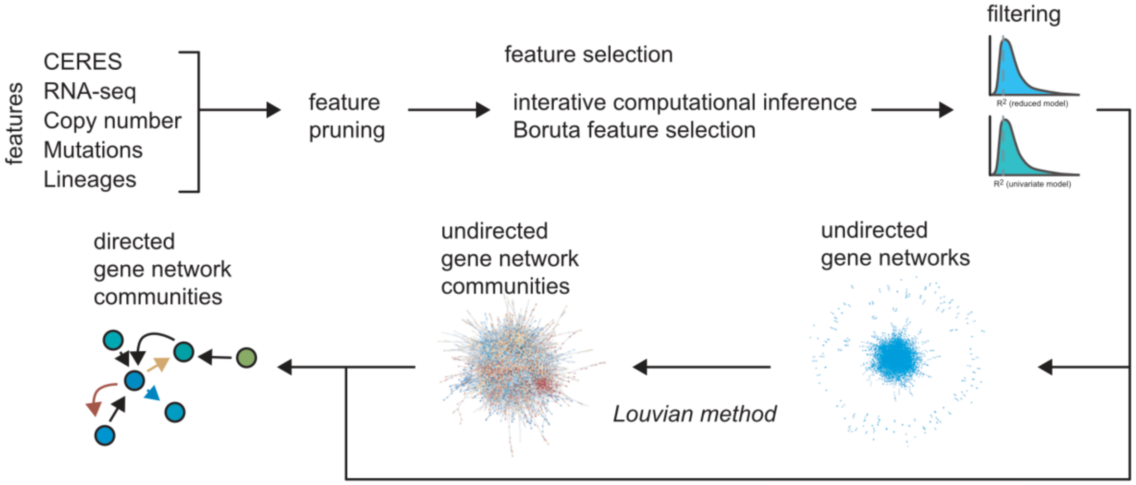

Supplementary Figure 3. Schematic of model and network building. Data sources (CERES scores, RNA-seq, copy number, mutations, and lineage) are used as features. Prior to model building, invariant/low variant features and non-expressed genes are pruned. The features are selected via an iterative processed followed by Boruta feature selection. Models are fit to the final reduced set of features and further filtered based on model scores. The feature-target genes were aggregated into an undirected gene network, of which communities are identified using the Louvain method. The communities are defined as directed networks based on the feature-target gene relationships. See Methods for more details.

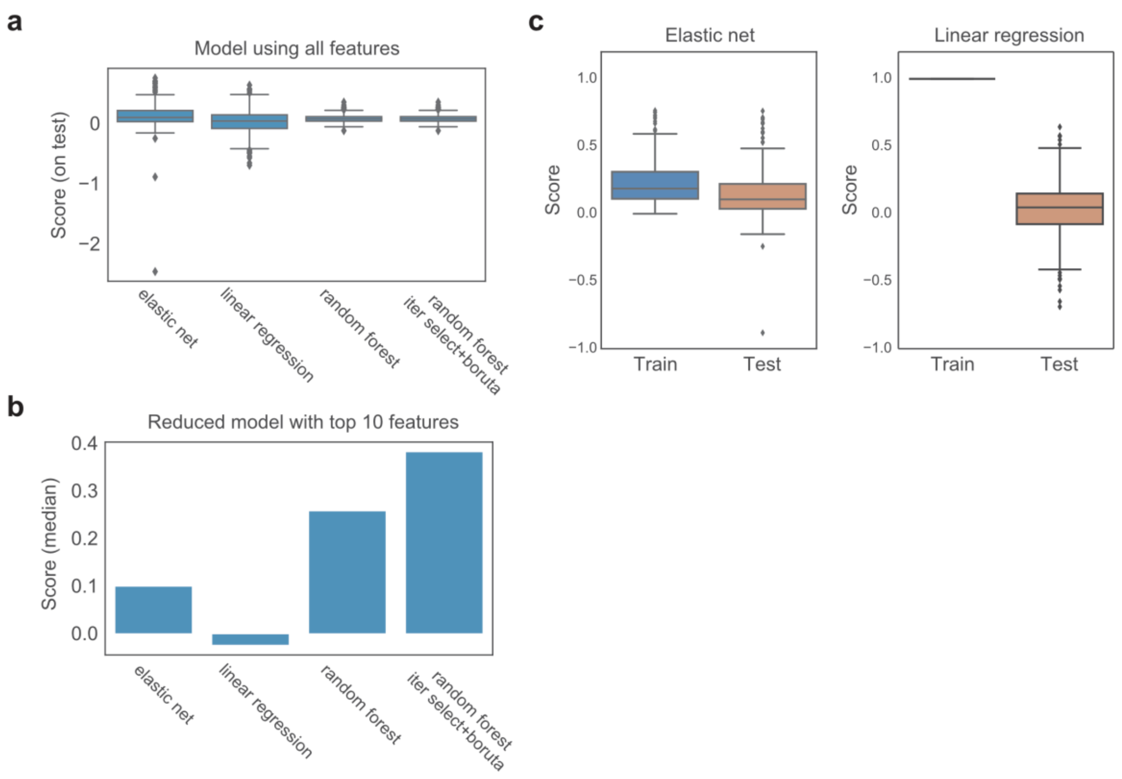

Supplementary Figure 4. Comparison in performance across different machine learning algorithms. (a) Comparison of model score (R2) on test set using all features based on different ML algorithms: elastic net, linear regression, random forest, or random forest with iterative/Boruta selection. (b) Comparison of model scores across different ML algorithms built based on the top 10 features. (c) Model score for train and test set for elastic net (left) and linear regression (right) models based on all features. For the box plots in (a) and (c), box shows the quartiles, whiskers indicate the maximum/minimum non-outlier observations, dots indicate outliers falling outside of 1.5 times interquartile range, and center is the median. There were n = 583 target genes, corresponding to 583 models per ML algorithm. Source data are provided as a Source Data file.

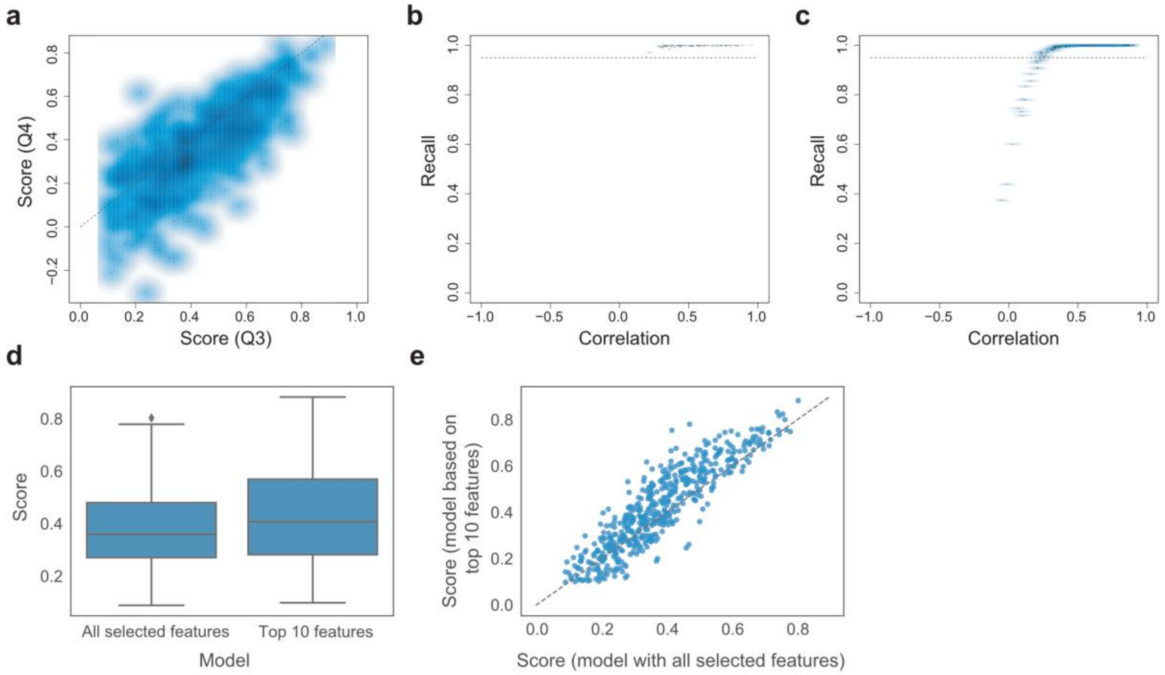

Supplementary Figure 5. Validation of model performance. (a) Scatter plot of model scores (R2s) on the 19Q4 vs 19Q3 test sets. Models are trained on the 19Q3 training set. (b-c) Recall versus correlation for 19Q3 (b) and 19Q4 (c) test sets. (d-e) Top 10 features are sufficient and shows comparable model performance to models with all significant features, as shown by box-whisker plot (d) and scatter plot (e). For the box plot in (d), box shows the quartiles, whiskers indicate the maximum/minimum non-outlier observations, dots indicate outliers falling outside of 1.5 times interquartile range, and center is the median. Validation was performed on n = 529 target genes/models after filtering (see Methods for more details). Source data are provided as a Source Data file.

a

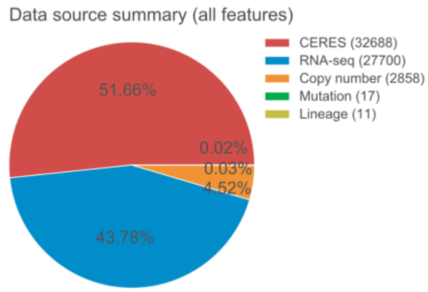

b

Feature	Feature source	Target gene
HAEMATOPOIETIC_AND_LYMPHOID_TISSUE	Lineage	PPCDC
PANCREAS	Lineage	KRAS
HAEMATOPOIETIC_AND_LYMPHOID_TISSUE	Lineage	NAMPT
SKIN	Lineage	SOX10
SKIN	Lineage	DUSP4
HAEMATOPOIETIC_AND_LYMPHOID_TISSUE	Lineage	ATP1B3
HAEMATOPOIETIC_AND_LYMPHOID_TISSUE	Lineage	CBFB
LARGE_INTESTINE	Lineage	CTNNB1
HAEMATOPOIETIC_AND_LYMPHOID_TISSUE	Lineage	NCKAP1
SKIN	Lineage	BRAF
SKIN	Lineage	ZEB2

c

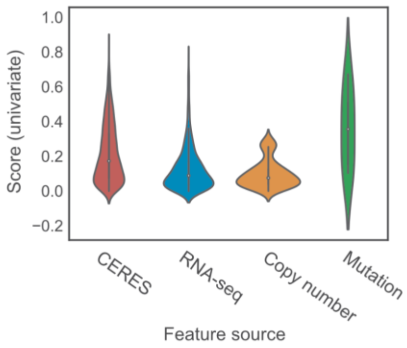

Supplementary Figure 6. Model feature summaries. (a) Classification of all features of all models by data source. (b) Significant lineage features and their target gene. (c) Univariate model scores as violin plots grouped by the data source of features. Source data are provided as a Source Data file.

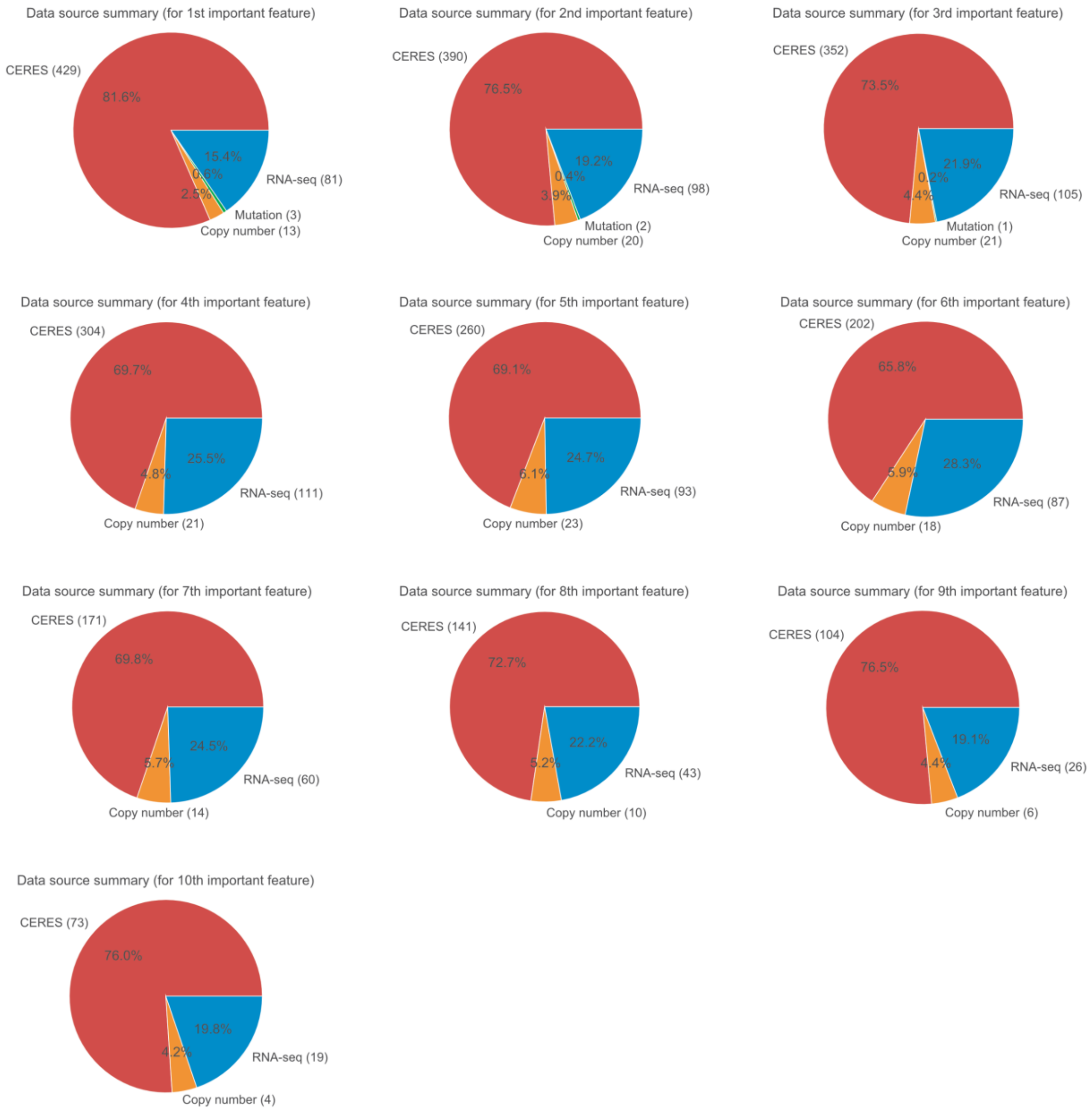

Supplementary Figure 7. Contribution of data sources for top 10 features. Pie chart showing breakdown of the data source type of features contributing to model prediction, for each nth most important feature. Source data are provided as a Source Data file.

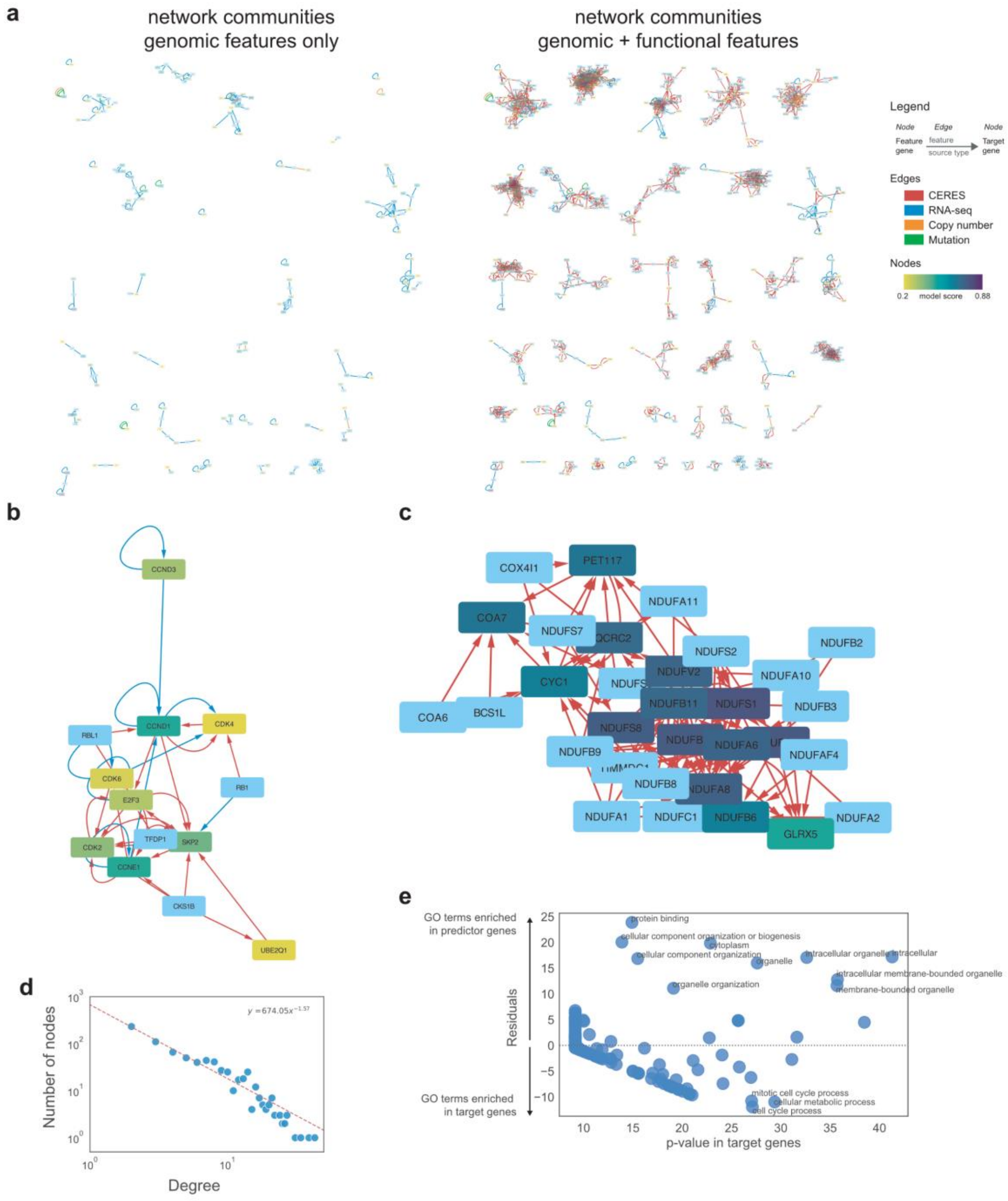

Supplementary Figure 8. Selected network communities. (a) All Louvain communities for networks with genomic features versus genomic + CERES features are shown in A. Networks for *CDK4* (b) and mitochondrial (c) extracted from model/network building. Nodes denote genes and edges denote feature-target gene relationships. Node colors are based on the score of the top 10 feature model of the corresponding gene as target. (d) Depicts the number of nodes having a given degree in the network which is then fit to a power law. (e) Residual plot identifies GO terms that are more (residuals of -log10 p-values>10) or less (residuals of -log10 p-values <-10) enriched in common architecture genes than in context specific genes. Dots represent the 100 most significant terms in target and predictor g:Profiler analysis result. If a term was among Top100 in one analysis but not in another, it was assigned the least significant p-value of the Top 100 terms in the analysis that the term is absent from. The p-values from g:Profiler are based on hypergeometric tests with multiple testing corrections using the g:SCS method. Source data are provided as a Source Data file.

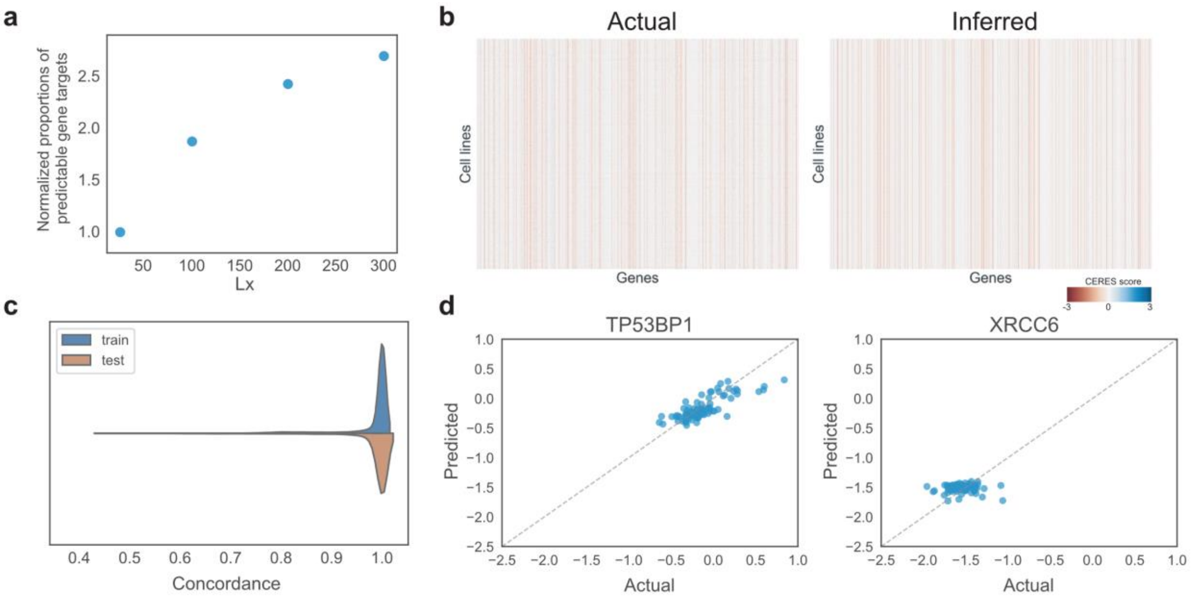

Supplementary Figure 9. Validation of lossy compression. (a) Saturation analyses based on lossy L25, 100, 200, and 300 lossy gene sets. Y-axis shows the normalized proportion of predictable gene targets, where predictable is defined as target genes with recall greater than 0.95. (b) Genome-wide comparison between actual and inferred CERES scores on the training set. (c) Concordance between actual and predicted CERES scores for train and test sets. (d) Scatter plot of predicted versus actual for selected genes *TP53BP1* and *XRCC6*. Source data are provided as a Source Data file.

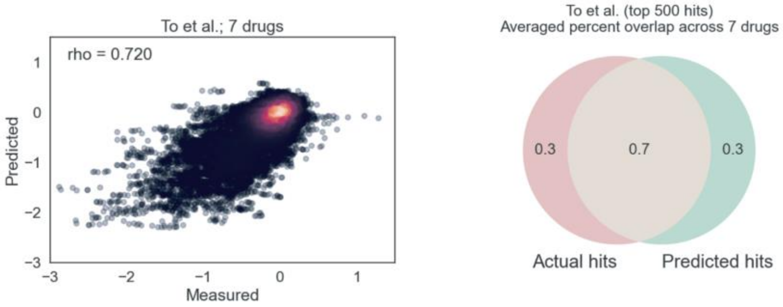

Supplementary Figure 10. To *et al.* chemical genomics hits are predicted by lossy compression sets. Seven different screens are graphed as a scatter plot. Predicted vs. Measured values for genome wide sgRNAs are graphed alongside a Venn diagram depicting the shared 'hits' in the top 500 differentially required genes. Source data are provided as a Source Data file.
