# Supplementary material for: A pan-CRISPR analysis of mammalian cell specificity identifies ultra-compact sgRNA subsets for genome-scale experiments
Source: Nat Commun. 2022 Feb 2;13:625. doi: 10.1038/s41467-022-28045-w (PMC8810922; doi:10.1038/s41467-022-28045-w)
Supplement: Supplementary file 2 — Description of Additional Supplementary Files [file 41467_2022_28045_MOESM2_ESM.pdf]

Description of Additional Supplementary Files

File Name: Supplementary Data 1
Description: This contains the GO ontology raw lists for the context specific genes.

File Name: Supplementary Data 2
Description: This contains the GO ontology raw lists for the predictive features.

File Name: Supplementary Data 3
Description: This contains the GO ontology raw lists for the L200.
